# Efficacy of ceftiofur *N*-acyl homoserine lactonase niosome in the treatment of multi-resistant *Klebsiella pneumoniae* in broilers

**DOI:** 10.1007/s11259-023-10161-7

**Published:** 2023-07-10

**Authors:** Reham A. Hosny, Zeinab A. El-badiea, Dalia M. A. Elmasry, Mai A. Fadel

**Affiliations:** 1 Reference Laboratory for Veterinary Quality Control On Poultry Production, Animal Health Research Institute, Agriculture Research Center, Giza, Egypt; 2 Nanomaterials Research and Synthesis Unit, Animal Health Research Institute, Agriculture Research Center, Giza, Egypt; 3 Pharmacology and Pyrogen Unit, Department of Chemistry, Toxicology, and Feed Deficiency, Animal Health Research Institute, Agriculture Research Center, Giza, Egypt

**Keywords:** Quorum, Quenching, Lactonase, *Klebsiella*, Niosome, Ceftiofur

## Abstract

In this study, the efficiency of the ceftiofur *N*-acyl homoserine lactonase niosome against multi-resistant *Klebsiella pneumoniae* in broilers was evaluated. Fifty-six *K. pneumonia*e isolates previously recovered from different poultry and environmental samples were screened for the *ahlK* gene. The lactonase enzyme was extracted from eight quorum-quenching isolates. The niosome was formulated, characterized, and tested for minimal inhibitory concentration (MIC) and cytotoxicity. Fourteen-day-old chicks were assigned to six groups: groups Ӏ and П served as negative and positive controls, receiving saline and *K. pneumoniae* solutions, respectively. In groups Ш and IV, ceftiofur and niosome were administrated intramuscularly at a dose of 10 mg/kg body weight for five consecutive days, while groups V and VI received the injections following the *K. pneumoniae* challenge. Signs, mortality, and gross lesions were recorded. Tracheal swabs were collected from groups П, V, and VI for counting *K. pneumoniae.* Pharmacokinetic parameters were evaluated in four treated groups at nine-time points. The niosome was spherical and 56.5 ± 4.41 nm in size. The viability of Vero cells was unaffected up to 5 × MIC (2.4 gml^−1^). The niosome-treated challenged group showed mild signs and lesions with lower mortality and colony count than the positive control group. The maximum ceftiofur serum concentrations in treated groups were observed 2 h following administration. The elimination half-life in niosome-treated groups was longer than that reported in ceftiofur-treated groups. This is the first report of the administration of *N*-acyl homoserine lactonase for the control of multi-resistant *K. pneumoniae* infections in poultry.

**Supplementary Information:**

The online version contains supplementary material available at 10.1007/s11259-023-10161-7.

## Introduction

*Klebsiella pneumoniae* is an opportunistic Gram-negative bacterial pathogen that normally inhabits the mucosal surfaces of humans and animals, and is also commonly found in different environmental sources such as water and soil (Struve and Krogfelt 2004; Hosny and Fadel 2021). It causes a wide variety of community-acquired infections in human, including urinary tract infections, pneumonia, septicemia, and liver abscesses (Hayati et al. 2019). *K. pneumoniae* is also found in livestock and poultry; causing respiratory symptoms, production loss, and even deaths if untreated or immunosuppressed (Hamza et al. 2016; Hayati et al. 2019). *K. pneumoniae* is one of the most common causes of yolk sac infections in chicks (Khan et al. 2004; Hosny and Fadel 2021). *K. pneumoniae* have been isolated from chickens suffered from omphalitis, dermatitis, cellulitis, and inflamed respiratory mucosa (Kowalczyk et al 2022). The general isolation rates of the bacteria in poultry are 10% to 35% (Aly et al 2014; Hamza et al. 2016; Hosny and Fadel 2021; Khelfa and Morsy 2015; Zhai et al. 2020).

The expression of virulence factors such as capsular polysaccharides, lipopolysaccharides, adherence factors, siderophore production, and biofilm formation have been reported to be under the regulation of quorum sensing in *Klebsiella* species (Clegg and Murphy 2017; Santajit et al. 2022). *K. pneumoniae* has the typical LuxR/LuxI quorum sensing system and the most commonly produced signal molecules is *N*-acyl-homoserine lactones (AHLs) (Chan 2013). It was reported that the most quantifiable AHLs produced by *K. pneumoniae* isolated from poultry samples were C4-HSL, C6-HSL, and C12-HSL (Hosny and Fadel 2021).

Beta-lactams are the most commonly used antibiotics for treating infections caused by Enterobacteriaceae in the veterinary sector (Rubin and Pitout 2014). Ceftiofur is a third-generation cephalosporin antibiotic with a wide spectrum of activity against Gram-negative and Gram-positive pathogens, that exerts its bactericidal action by inhibition of the bacterial cell wall synthesis (El-Sayed et al. 2015). It has worldwide approval for respiratory diseases in chicks and turkey poults (Hornish and Katarski 2002). *K. pneumoniae* resistance to broad-spectrum β-lactams has become an alarming and growing public health challenge based on the recent reports on antimicrobial resistance from the US Centers for Disease Control and Prevention (CDC) and the European Centre for Disease Prevention Control (CDC 2019; ECDC 2020). The increasing incidence of multidrug-resistant bacteria poses a significant challenge for physicians and veterinarians, limiting treatment choices and raising concerns about transmission to humans through the food chain and the emergence of super-resistant bacteria (Li et al. 2022).

Since quorum sensing regulates different biological functions associated with virulence, and as the emergence of multi-drug resistant bacterial strains is on the rise, there is increasing pressure to discover new antimicrobial agents targeting virulence, including bacterial adhesion, toxin function, regulation of virulence expression (D'Angelo et al. 2018).

Quorum quenching (QQ) is the process of interrupting quorum sensing through three mechanisms: (1) interference with the synthesis of signal molecules, (2) enzyme-mediated degradation of signal molecules, and (3) blocking of signal molecule receptors (Geske et al. 2008; Galloway et al. 2011). To date, four different types of QQ enzymes have been identified, including, AHL-lactonase, AHL-acylase, oxidoreductase, and paraoxonase (Torabi Delshad et al. 2018).

The AiiA enzyme was the first enzyme identified in a *Bacillus* species strain and expressed in the agricultural pathogen *Erwinia carotovora*, causing a reduction in virulence (Reina et al. 2022). Subsequently, many AHL-degrading enzymes, such as AhlD, AiiM, AidC, and MomL, were identified in *Arthrobacter,* *Microbacterium*, *Chryseobacterium,* and *Muricauda*, respectively (Cai et al. 2018). Many AHL-degrading QQ bacteria, such as *Klebsiella*, *Pseudomonas*, *Ralstonia*, *Variovorax*, *Comamonas,* and *Agrobacterium* genera were isolated from a wide range of aquatic animals and environments (Chan 2013; Chen et al. 2013). In *K. pneumoniae,* AhlK encodes an AHL-degrading enzyme, and the main two AHLs degraded by AhlK were C6-HSL and 3-oxo-C6-HSL (Park et al. 2003; Chan 2013). Previous studies have displayed that QQ strains can be applied as biocontrol agents in aquaculture and agriculture (Dong et al. 2000; Torres et al. 2016; Zhang et al. 2019; Chen et al. 2020). However, little is known about their potential use as biocontrol agents in the poultry sector.

Although the approach against virulence factors has become a promising strategy for combating different bacterial pathogens, one of its limitations is its inability to completely eradicate the infection (Dehbanipour and Ghalavand 2022). This limitation is overcome by combining antivirulence drugs with antibiotics (Bortolotti et al. 2019; Zhao et al. 2020; Dehbanipour and Ghalavand 2022). Furthermore, traditional drug delivery systems for these therapeutic compounds have significant disadvantages, including a lack of compatibility at the required level, poor biodistribution, disturbed release, and limited accuracy to reach the target sites (Gao et al. 2018). Recently, the development of nanoparticles for drug delivery had a significant impact on the pharmacokinetic profile and therapeutic index of drugs (Gao et al. 2018). Niosomes, defined as non-ionic surfactant-based liposomes, are one of several nanoparticle delivery systems that offer several advantages over other delivery systems through enhancing drug solubility, immune evasion, modulating drug release, and delivering drug molecules to target sites (Kumar and Rajeshwarrao 2011). They are distinguished from other nanoparticles due to their ability to incorporate both hydrophilic and hydrophobic drugs, proteins, enzymes, and genes directly into infection sites (Kumar and Rajeshwarrao 2011; Ag Seleci et al. 2016; Ge et al. 2019).

On this basis, QQ *K. pneumoniae* isolates previously recovered from different poultry and environmental samples were identified and characterized with the aim of formulating and characterizing the extracted AhlK lactonase enzyme in a ceftiofur *N*-acyl homoserine lactonase niosome form to be assessed in the control of multi-resistant *K. pneumoniae* infection in broilers.

## Material and methods

### Identification of *K. pneumoniae* isolates

A total of 56 K*. pneumoniae* isolates were previously recovered from eggs, organs, cloacal swabs, and different environmental niches collected from different markets, farms and backyards with history of enteritis in Giza governorate and summarized as follows: (eight from chicken eggs, four from duck eggs, one from geese egg, four from pigeon eggs, ten from chicken organs, four from duck organs, eight from duck cloacal swabs, two from pigeon cloacal swabs, one from water, two from chicken litter, twelve from duck litter) (Hosny and Fadel 2021). The identification was done as described by (Hamza et al. 2016; Hosny and Fadel 2021) using buffered peptone water (Oxoid Limited, Thermo Fisher Scientific Inc., UK) and Mac-Conkey agar plates (Oxoid Limited, Thermo Fisher Scientific Inc., UK). The plates were examined for mucoid and pink colonies growth. Confirmation of the *K. pneumoniae* isolates was done as described by (Hansen et al. 2004) using the API20E system (BioMerieux, Marcy l’Etoile, France) present in the Reference Laboratory for Veterinary Quality Control on Poultry Production, Animal Health Research Institute, Dokki, Egypt (RLQP, Egypt).

### Screening of* K. pneumoniae* quorum quenching gene *ahlK* by conventional PCR

The DNAs of 56 K*. pneumoniae* isolates were extracted using a QIAGEN kit (Qiagen, Germany, GmbH). The *ahlK* gene was amplified using forward and reverse primers: KgInF, 5-GCACTCTGATCATACGGGAGCAAT-3 and KgInR, 5- TGGCCCGGTGAATGCCCTGGGGTG-3. The amplification was conducted according to Chan (2013) in a Thermal Cycler (Applied biosystem 2720). Each PCR reaction was performed in 25 µL total reaction volume containing 12.5 µL of Emerald Amp Max PCR Master Mix (Takara, Japan), 1 µL of each primer with a concentration of 20 pmol, and 5 µL of genomic DNA extract 5.5 µL of DNAse and RNAse free water. The amplification conditions were as follows: initial denaturation at 95 °C for 5 min, followed by 40 cycles for denaturation at 95 °C for 45 s, annealing at 65 °C for 45 s, extension at 72 °C for 1 min, and a final extension at 72 °C for 10 min. The products of PCR were separated by electrophoresis on 1.5% agarose gel (Applichem, Germany, GmbH) in 1 × TBE buffer at room temperature. The expected molecular weight for the electrophorized product is 250 bp.

### Sequencing of *ahlK *gene from *K. pneumoniae* isolates

The PCR products for the selected six QQ isolates containing *ahlK* gene recovered from duck were subsequently purified by a QIAquick Gel Extraction Kit (Qiagen, Germany, GmbH). The purified PCR products (30 ng/μl) were sequenced in RLQP, Egypt using a BigDye Terminator V3.1 cycle sequencing kit (Applied Biosystems, Foster City, CA) and DNA Analyzer 3500 XL (Applied Biosystems, Tokyo, Japan). The alignment of the sequences was performed using BIOEDIT software. The Basic Local Alignment Search Tool (BLAST®) was used to compare the results of the *ahlK* gene sequencing to the gene bank database (NCBI) to establish identification at the isolate level.

### Extraction of *N*-acyl homoserine lactonase

The enzyme extract was prepared as described by (See-Too et al. 2017; Hosny and Fadel 2021). Briefly, eight QQ *K. pneumoniae* isolates containing *ahlK* gene were grown overnight in Luria broth agar plates (LB) (Oxoid Limited, Thermo Fisher Scientific Inc., UK) buffered with 50 mM 3-(N–morpholino) propane sulfonic acid (MOPS) (pH 6.5) (Santa Cruz Biotechnology, Inc, Canada). The isolates were ultracentrifuged and the supernatant was extracted using acidified ethyl acetate (0.1%, v/v glacial acetic acid) (Fischer Scientific, Leicestershire, UK). The residues were dissolved in deionized water and used for biosensor and chromatographic assays.

### Screening of quenching activity of *N*-acyl homoserine lactonase extract

#### *A*. *tumefaciens* NTL4 biosensor

The quenching activity of the *N*-acyl homoserine lactonase extract was assayed using a well diffusion assay as described by (Torres et al. 2013; Torabi Delshad et al. 2018; Hosny and Fadel 2021). The enzyme extract was grown in a 10 ml Tryptic soy broth (TSB) supplemented with 5 mg l^−1^ AHLs for 24 h at 20 °C with constant agitation (120 xg). The synthetic AHLs used were as follows: *N*-butyryl-DL-homoserine lactone (C4-HSL), *N*-hexanoyl-DL-homoserine lactone (C6-HSL), *N*-(3-oxo)- hexanoyl-DL-homoserine lactone (3-oxo-C6-HSL), *N*-heptanoyl-DL-homoserine lactone (C7-HSL), *N*-octanoyl-DL-homoserine lactone (C8-HSL), *N*-(3-oxo)-octanoyl-L-homoserine lactone (3-oxo-C8-HSL), *N*-decanoyl-DL-homoserine lactone (C10-HSL), *N*-dodecanoyl-DL-homoserine lactone (C12-HSL), and *N*-tetradecanoyl-DL-homoserine lactone (C14-HSL) (Santa Cruz Biotechnology, Inc, Canada). The mixtures were streaked on LB agar plates containing 60 µg/ml of 5-Bromo-4-chloro-3-indolyl-β-D-galactopyranoside (X-gal) (Sigma-Aldrich, Egypt) seeded with an overnight-grown *A. tumefaciens* NTL4(pZLR4) strain. The biosensor strain was previously grown in LB agar plates supplemented with 150 µg/ml of gentamicin (Sigma-Aldrich, Egypt) and 0.5%, w/v glucose (Sigma-Aldrich, Egypt) at 28^◦^C (Hosny and Fadel 2021). The same concentration of each AHL was added to 10 ml of cell-free TSB medium as negative controls, and processed under similar conditions. The plates were then incubated at 28^◦^C for 48 h to check the capability of isolates to inhibit blue pigment production.

#### Reverse phase high-performance liquid chromatography

##### Chemicals and reagents

Acetonitrile (ACN) and methanol (MeOH) were obtained from Fischer Scientific (Leicestershire, UK). Ethylenediaminetetraacetic acid (EDTA) was purchased from Merck (Darmstadt, Germany). De-ionized water of HPLC grade was obtained using a Milli-Q system (Waters Corp., Milford, MA, USA).

##### Standards

Two *N*-acyl-homoserine Lactone standards were used, including *N*-hexanoyl-DL-homoserine lactone (C6-HSL) and *N*-(3-oxo)-hexanoyl-DL-homoserine lactone (3-oxo-C6-HSL).

##### Preparation of AHL standards

The AHL standards stock solutions were prepared as described by (Hosny and Fadel 2021) by dissolving AHL standards in acetonitrile to obtain a concentration of 1mgml^−1^. The calibration standard concentrations were diluted from stock solution at a range from 5 to 50 µgml^−1^.

##### Chromatographic assay

Analysis was determined as described by Hosny and Fadel (2021) using an Agilent 1200 series (Agilent Co., Santa Clara, CA, USA) equipped with a quaternary pump, vacuum degasser autosampler injector, and ultraviolet detector. To analyze AHL-degradation activity, 10µgml^−1^ of C6-HSL and 3-oxo-C6-HSL were incubated with the extract at 20 °C in buffered LB and aqueous media (0.07% NaCl) with MOPS (pH 6.5) for 0 and 24 h. The C6-HSL and 3-oxo-C6-HSL in buffered LB and aqueous media were used as negative controls. HPLC analysis was performed using optimized chromatographic parameters outlined in Table 1 to obtain high separation efficiency.

**Table 1:** Chromatographic conditions used in the detection of *N*-acyl homoserine lactonase and ceftiofur using HPLC method

Parameters	*N*-acyl homoserine lactonase (Hosny and Fadel 2021)	Ceftiofur (Abd Elhafeez and Fadel 2021)
Ultraviolet detector	210 nm	267 nm
Column Temperature	35 °C	40 °C
Column	Agilent C18 column (4.6-mm internal diameter, 150 mm, 5-μm particle size)	
Mobile phase	A gradient mobile phase of methanol: water (35:65, v/v) was used for 5 min, followed by methanol: water (80:20, v/v) for 10 min. After that, 65% methanol: water was used for 15 min. Finally, methanol (35:65, v/v) was used for 8 min	Isocratic mobile phase of Deionized water containing 0.1% TFA and ACN (90:10, v/v)
Flow rate	1 ml/min	
Injection volume	10 µl	

##### Validation of the HPLC method

The validation was performed according to (ICHQ2(R1) 2005; USP 2021) guidelines for different parameters such as linearity, range, recovery, accuracy, detection limit (DL), and quantification limit (QL).

##### Thermal and pH stability of *N*-acyl homoserine lactonase extract

The stability of the *N*-acyl homoserine lactonase against different thermal and pH conditions was assessed according to Sakr et al. (2013) with some modifications in the thermal temperatures and pH. Briefly, 1 mL of the enzyme extract was incubated in the water bath at 40, 60, and 90^∘^C for 60 min. The pH of the enzyme extract (1 mL) was adjusted at 4, 6, 8, and10 using a pH meter (Jenway, UK); the buffers used were 1 mol HCl (AppliChem, Darmstadt, Germany) and 1 mol NaOH (AppliChem). After heat and pH treatments, the quenching activity of the enzyme extracts was assayed against *A. tumefaciens* NTL4(pZLR4) biosensor strain using a well diffusion assay as previously described. Following 48 h of incubation, the plates were observed for the inhibition zones of pigment production.

### Preparation of ceftiofur *N*-acyl homoserine lactonase niosome

#### Chemicals and reagents

Ceftiofur sodium (Naxcel^®^) was obtained from Zoetis, USA in a form of a water-soluble powder, where each 1 mL contains 50 mL of ceftiofur base. Liposome-base (phospholipid) was purchased from Chemajet Chemical Company, Alexandria, Egypt. Tween 20 and phosphate-buffered saline (PBS) were obtained from (Sigma-Aldrich, St. Louis, USA) and de-ionized water.

#### Preparation

Two concentrations of ceftiofur *N*-acyl homoserine lactonase mixtures (75% v/25%v) and (50% v/50%v) (1 mg ml^‐1^) were prepared by mixing ceftiofur with lyophilized *N*-acyl homoserine lactonase extract in the pharmacology unit, Animal Health Research Institute (AHRI), Giza, Egypt.

Ceftiofur *N*-acyl homoserine lactonase niosome was prepared according to (Kazi et al. 2010; Rasti et al. 2012) in the Nanomaterial Research and Synthesis unit, AHRI, Giza, Egypt. Briefly, 0.0129 g of ceftiofur *N*-acyl homoserine lactonase (75% v/25%v) and (50% v/50%v) were solubilized in 10 ml of PBS solution as an aqueous phase. The solution was mixed with 10 ml of liposome suspension, 20 ml of tween 20, and 60 ml of deionized water. The mixture was subjected to sonication using a homogenous blender of 1000 watts at 25 °C for 10 min. The deionized water was slowly added to the oil-phase mixture, followed by sonication for 30 min.

#### Determination of minimum inhibitory concentration (MIC) and minimum bactericidal concentration (MBC) for ceftiofur, *N*-acyl homoserine lactonase, and ceftiofur* N*-acyl homoserine lactonase niosome using broth macro-dilution method

The broth macro-dilution technique was used to determine the MIC and MBC for ceftiofur, *N*-acyl homoserine lactonase, and ceftiofur *N*-acyl homoserine lactonase niosome (75% v/25%v) and (50% v/50%v) following Clinical and Laboratory Standards Institute guidelines (CLSIM07-A9 2012; CLSI 2017). Two-fold serial dilutions of the tested solutions (1mgml^−1^) in distilled water were prepared. The selected range to define the *K. pneumoniae* organism as resistant (resistance breakpoint ≥ 8) according to (CLSIVET01S 2015). The medium used in the macro-dilution testing is Mueller–Hinton broth (MHB) (Oxoid Limited, Thermo Fisher Scientific Inc., UK). The *K. pneumoniae* isolate (KP19) used was previously isolated from a chicken organ that was an AHL producer (C4, C6, C8, and C12), resistant to cefotaxime, cefaclor, cefoxitin, tetracycline, gentamycin, and amoxicillin-clavulanic acid (Hosny and Fadel 2021). Furthermore, the KP19 isolate was ESBL and AmpC producer as it produced positive amplification for *blaTEM, blaSHV, and AmpC* genes (data not shown). For bacterial inoculum preparation, pure mucoid colonies cultured overnight on MacConkey medium were suspended in MHB medium and adjusted to 5 × 10^5^ CFUml^−1^. The bacterial inoculum (0.1 ml) was added to 2 ml of a liquid MHB medium containing ceftiofur, *N*-acyl homoserine lactonase**,** and ceftiofur *N*-acyl homoserine lactonase niosome in the dilution series. The tubes were then incubated for 16 to 20 h at 35 ± 2 °C. The last two tubes contained positive control of *K*. *pneumoniae* bacteria and negative control of ceftiofur. The MIC was considered the lowest concentration of the tested solutions that produced no visible growth (no turbidity recorded). The MBC value was determined after subculturing of each tested dilution which showed no turbidity on to plate count agar media (Oxoid Limited, Thermo Fisher Scientific Inc., UK) for 16 to 20 h at 35 ± 2 °C. MBC was defined as the lowest concentration of the tested solutions that produced no bacterial growth on the agar plates.

#### Determination of synergy between the ceftiofur and *N*-acyl homoserine lactonase using checkerboard broth micro-dilution method

The fractional inhibitory concentration (FIC) of combined ceftiofur and *N*-acyl homoserine lactonase was determined by a checkerboard broth micro-dilution method as described by (Fadel et al. 2022). The fractional inhibitory concentration (FIC) was calculated as follows: FIC of *N*-acyl homoserine lactonase = MIC of *N*-acyl homoserine lactonase in combination with ceftiofur / MIC of *N*-acyl homoserine lactonase alone, FIC of ceftiofur = MIC of ceftiofur in combination with *N*-acyl homoserine lactonase / MIC of ceftiofur alone and FIC index (FICI) = FIC of *N*-acyl homoserine lactonase + FIC of ceftiofur. Synergism is defined as FICI < 0.5, additive effect as FICI 0.5–1, indifference as FICI = 1–4, and antagonism as FICI > 4.

#### Characterization of ceftiofur *N*-acyl homoserine lactonase niosome

The niosome (50% v/50%v) was characterized according to (Kazi et al. 2010; Rasti et al. 2012) using Transamination electron microscopy and Zetasizer. Transamination electron microscopy (TEM) (JSM-6400, JEOL, Tokyo, Japan) was used to analyze the shape and size, while the Zetasizer (Nanotrac Wave II, Microtrac, USA) was used to measure the electrical conductivity, zeta potential, droplet size, and size distribution (polydispersity index, PDI).

#### Cytotoxicity of the ceftiofur *N*-acyl homoserine lactonase niosome

The cytotoxicity of the niosome (50% v/50%v) was evaluated according to (Vichai and Kirtikara 2006; Borin et al. 2018) in African green monkey kidney cells (Vero cell) using Sulforhodamine B assay (SRB). Briefly, 100 μL of the cell suspension (5 × 10^4^ cells ml^−1^) was added in 96-well plates (Corning Incorporated – Life Sciences, NY, United States) and incubated for 24 h. Cells were then treated with 100 μL of the niosome solution at various concentrations (0.01, 0.1, 1,10, 100 µgml^−1^) in triplicates. After 72 h, plates were washed twice with 1X PBS and the cells were fixed by 150 μL of 10% Trichloroacetic acid (TCA) (Fischer Scientific, Leicestershire, UK) and incubated at 4 °C for 1 h. The TCA solution was removed, and the cells were washed 5 times with distilled water. After that, 70 μL of SRB solution (0.4% w/v) was added and incubated in a dark place at room temperature for 10 min. Plates were then washed 3 times with 1% acetic acid and allowed to air-dry overnight. Finally, 150 μL of Tris- EDTA buffer (10 mM) was added to dissolve the protein-bound SRB stain and the absorbance was measured at 540 nm using a BMG FLUOstar Omega microplate reader (BMG LABTECH, Ortenberg, Germany). Unexposed cells were used as negative controls. The absorbance values were represented as mean ± standard deviation (SD). The cell viability was calculated according to the following equation (Vatan 2022): Cell viability % = 1- (A _experimental_/A _control_) × 100, where A _experimental_ is the absorbance of the cells exposed to the niosome, and A _control_ is the absorbance of the negative control. The half-maximal inhibitory concentration (IC_50_) was obtained from the fitting of the dose–response curve as described by Ihling et al. (2022).

#### Efficacy of ceftiofur *N*-acyl homoserine lactonase niosome in the treatment of multi-resistant *K. pneumoniae* in broilers

##### Materials

The present study was executed using ceftiofur sodium Naxcel® (Zoetis, USA). Ceftiofur and Ceftiofur *N*-acyl homoserine lactonase niosome were administered for birds intramuscularly (I.M) into the gluteal muscles once daily for five consecutive days at a dose of 10 mg/kg body weight (BW) (El-Sayed et al. 2015). The reference standards of ceftiofur sodium VETRANAL ≤ 100% and desfuroylceftiofur were provided by Sigma-Aldrich (St. Louis, MO, USA) and Toronto Research Chemical (Ontario, Canada), respectively. The stock solutions of standards were prepared as described by Abd Elhafeez and Fadel (2021) by dissolving standards in water to obtain a concentration of 1mgml^−1^. The calibration standard concentrations were diluted from stock solution at a range from 0.05 to 10 µgml^−1^. The quality control samples were prepared at three levels in blank chicken serum to be 0.1, 1, and 10 µgml^−1^.

Isooctane was obtained from Fischer Scientific (Leicestershire, UK). 5% Trichloroacetic acid (TCA) was obtained from Fischer Scientific (Leicestershire, UK). Spectrophotometric grade trifluoroacetic acid (TFA) ≥ 99.9% and ammonium acetate buffer 0.05 M; pH5 were purchased from Sigma-Aldrich (St. Louis, MO, USA). Acetonitrile (ACN), methanol (MeOH), trichloroacetic acid (TCA), and de-ionized water of HPLC grade.

##### Bacterial inoculum

The *K. pneumoniae* strain (KP19) used in the experimental study was the same one used in the determination of MIC and MBC (Hosny and Fadel 2021).

##### Experimental birds and facilities

A total of 180 commercial 14-day-old Ross broilers were purchased from a commercial hatchery in Behera. Birds were housed in battery cages in a biosafety level Π experimental room in the RLQP, Egypt, which has temperature, lighting, and ventilation controls according to Ross Broiler management specifications (Aviagen 2018). Birds were provided with ad-libitum water and a grower diet according to Ross 308 broiler Nutrition specifications guidelines (Aviagen 2014). Tracheal and environmental swabs were taken from chicks (extra than 180), experimental room, and cages before the housing of chicks and examined for *K. pneumoniae* infection.

##### Experimental design

The experimental design of this study was conducted according to (El-Sayed et al. 2015; Tantawy et al. 2018) with a modification in the age of birds. Chickens were divided into six groups of three replicates (10 birds /group): group Ӏ was received 1 ml of saline solution and assigned as a negative control, group П served as a positive control group that was challenged orally with 1 ml of *K. pneumoniae* isolate (KP19) containing 10^9^ CFU/ml on day 15. Groups Ш and IV were administrated with ceftiofur and niosome (10 mg/kg BW, I.M), respectively on days 16^th^ to 20^th^. Groups V and VI were challenged orally with *K. pneumoniae* culture containing 10^9^ CFU/ml on day 15 followed by administration of ceftiofur and niosome, respectively (10 mg/kg BW, I.M) on days 16^th^ to 20^th^.

##### Samples

Clinical signs and mortality were recorded twice daily (09:00 a.m. and 17:00 p.m.). Tracheal swabs were collected from groups П, V, and VI on days 16–21 for the counting of *K. pneumoniae* (Table S1)*.* Blood samples were collected from the wing veins of five birds from groups Ш and IV, V, and VI at nine-time points (0.16, 0.5, 1, 2, 4, 8, 10, 12 and 24 h) following administration of ceftiofur and noisome on day 16 for studying of pharmacokinetic (PK) parameters (Table S1). Blood samples were subsequently centrifuged for 10 min at 2000 rpm and stored at − 20 °C until assayed. Birds were euthanized on day 21 and samples were taken from lung, liver, spleen, and intestine to examine gross lesions (Table S1).

##### Quantification of ceftiofur in serum samples

The concentration of ceftiofur in serum samples was determined by the HPLC method using an Agilent 1200 series (Agilent Co., Santa Clara, CA, USA) at AHRI, Giza, Egypt as described by Abd Elhafeez and Fadel (2021). Ceftiofur was extracted as described by Abd Elhafeez and Fadel (2021). Briefly, 200 µL of quality control and serum samples were mixed with 100 µL of 5% TCA. The mixtures were centrifuged for 10 min at 4000 × g and the clear aqueous phase was transferred into HPLC vials. The chromatographic conditions were outlined in Table 1, and validation was performed according to (ICHQ2(R1) 2005; USP 2021) guidelines.

##### Assessment of PK parameters

Mean serum levels of ceftiofur versus the time course were investigated using a compartmental approach (El-Sayed et al. 2015). The pharmacokinetic parameters, including maximum serum drug concentration (Cmax), absorption half-life t_1/2 ka_, elimination half-life (t1/2), apparent volume of distribution (V/F), apparent total clearance of the drug from serum CL/F, time to reach maximum serum concentration (T_max_), area under the concentration–time curve from 0 to 24 h (AUC_0−24_), mean residence time (MRT) were calculated according to Zhang et al. (2010) using pk solver an add-in program for Microsoft Excel, version 2.

## Statistical analysis

Statistical analysis was carried out using IBM SPSS Statistics (version. 21.0. Armonk, NY: IBM Corp.). Kolmogorov–Smirnov and Shapiro–Wilk tests were used to check the normality of the data of bacterial count and pharmacokinetic parameters. A Kruskal–Wallis’s test was used to determine the differences in the bacterial count between ceftiofur and niosome treated challenged groups and the positive control group. A Mann–Whitney test was used to determine the difference between the ceftiofur and niosome treated challenged groups. An independent sample t-test was used to examine the significance of differences in the pharmacokinetic parameters of ceftiofur in four treated groups*.* The *P*-values were considered significant at values ≤ 0.05.

## Results

### Screening of* K. pneumoniae* quorum quenching gene *ahlK* by conventional PCR

PCR analysis of 56 K*. pneumoniae* isolates revealed the detection of *ahlk* gene in 14.3% (8/56) of isolates at 278 bp as follows: duck cloacal swabs (KP33, KP39), duck litters (KP42, KP45, KP46, KP50, KP52), and water (KP56) (Table 2). All eight isolates exhibited resistance to cefotaxime (Hosny and Fadel 2021) (Table 2). Only two isolates were non-AHL producers (KP39 and KP50), while other six isolates were recorded as AHLs producers (KP33, KP42, KP45, KP46, KP52, and KP56) (Hosny and Fadel 2021) (Table 2).

**Table 2:** Screening of AHL-quenching activity of *N*-acyl homoserine lactonase extracts using *A*. *tumefaciens* NTL4 biosensor

Isolate number (Hosny and Fadel 2021)	Types of samples (Hosny and Fadel 2021)	Quorum quenching	Quorum sensing (Hosny and Fadel 2021)	Antibiotic resistance pattern (Hosny and Fadel 2021)
KP33	Duck swabs	3OXO-C6, C6	C4, C6, C8, C10	CTX -NA-CN
KP39	Duck swabs		negative	CTX -C-TE-AMC
KP42	Duck litters		C4, C7, 3OXO-C6, C6, C8, 3OXO-C8, C10, C12, C14	CTX
KP45	Duck litters		C4, C7, 3OXO-C6, C6, C8, 3OXO-C8, C10, C12, C14	CTX -CEC-NA
KP46	Duck litters		C4, C7, 3OXO-C6, C6, C8, 3OXO-C8, C10, C12, C14	CTX -CEC
KP50	Duck litters		negative	CTX
KP52	Duck litters		C4, C7, 3OXO-C6, C6, C8, 3OXO-C8, C10, C12, C14	CTX -IPM
KP56	Water		C4, C7, 3OXO-C6, C6, C8, 3OXO-C8, C10, C12, C14	CTX -CEC-TE-NA-CIP-CN

*CTX* Cefotaxime, *CEC* Cefaclor, *IPM* Imipenem, *AMC* Amoxicillin-clavulanic, *C* Chloramphenicol, *TE* Tetracycline, *NA* Nalidixic acid, *CIP* Ciprofloxacin, *CN* Gentamycin

### DNA sequencing and alignment of *ahlK* gene

The comparison of the *ahlK* sequences in the present study with other known AHL lactonase nucleotide data available in the GenBank database for *ahlK* revealed high similarity (up to 100%).

### Pairwise identity matrix of nucleotide and amino acid sequences

The pairwise identity matrix revealed a high degree of similarity among the six *K. pneumoniae* isolates (95% to 100%) to the amino acid data available in the GenBank database for *ahlK* group. These isolates were very similar to *K. pneumoniae* strain USA (accession number CP067563.1), *K. pneumoniae* strain China (accession number CP045193.1), and *K. pneumoniae* strain United Kingdom (accession number CP057459.1) (Fig. S1).

### Screening of AHL-quenching activity of *N*-acyl homoserine lactonase extract using *A*. *tumefaciens* NTL4 biosensor

All eight *K. pneumoniae* isolates (2 from duck cloacal swabs, 5 from duck litters, and 1 from water) were able to degrade C6-HSL and 3-oxo-C6-HSl producing inhibition zones with mean diameter sizes of 31 and 25, respectively; suppressing the activation of the indicator strain.

### Screening of AHL-quenching activity of *N*-acyl homoserine lactonase extract using reverse phase high-performance liquid chromatography

**Fig. 1:**
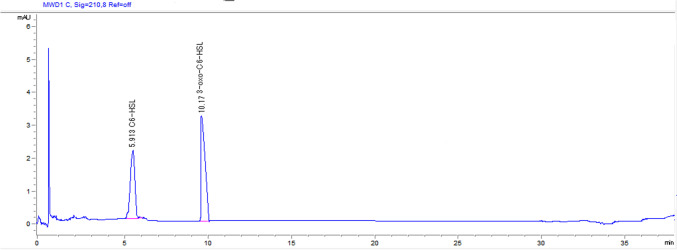
HPLC chromatogram of C6-HSL and 3-oxo-C6-HSL standards at a concentration of 5 µg/ml. C6-HSL and 3-oxo-C6-HSL standards expressed two peaks with retention times of 5.9, and 10.17 min, respectively

The method was accurate with high recovery for C6-HSL and 3-oxo-C6-HSL standards in a percentage of 93% and 111%, respectively. The standards displayed good linearity (˃ 0.99) with a low detection limit (LOD) and quantification limit (LOQ) as 1.9 and 1.4 μg/ml, respectively for LOD and 5.5 and 4.4 μg/L, respectively for LOQ. Specificity and selectivity of standards revealed retention times of 5.9 and 10.17 min, respectively (Fig. 1).

**Fig. 2:**
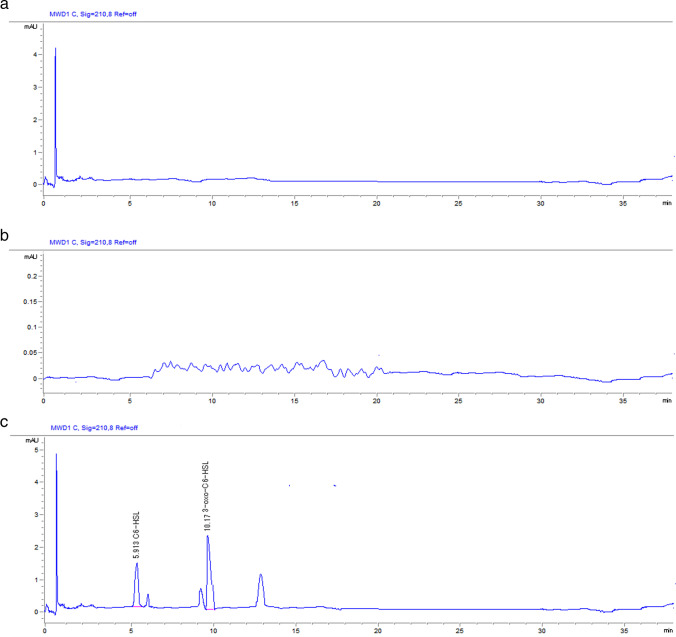
**a**,** b**, and** c**. HPLC chromatograms of C6-HSL and 3-oxo-C6-HSL degraded by *ahlK* lactonase activity. **A**, **B**) Complete degradation of C6-HSL and 3-oxo-C6-HSL chromatograms in **A**) an aqueous medium after incubation for 0 and 24 h and **B**) in LB medium after incubation for 24 h. **C**) Partial degradation of C6-HSL and 3-oxo-C6-HSL chromatograms in LB medium after incubation for zero hours

It revealed the complete disappearance of two AHL chromatograms (C6-HSL and 3-oxo-C6-HSL) following incubation of standards with lactonase extract in aqueous medium at 0 and 24 h and in LB medium at 24 h (Fig. 2a, b). Partial degradation of two AHL chromatograms (C6-HSL and 3-oxo-C6-HSL) was obtained following incubation of standards with lactonase extract in LB medium at zero hours (Fig. 2c). These findings suggested that *ahlK* lactonase has quenching activity towards C6-HSLs and 3-oxo-C6-HSLs.

### Thermal and pH stability of *N*-acyl homoserine lactonase

The enzyme retained its activity when incubated at 40^∘^C and 60^∘^C for 60 min evidenced by the presence of inhibition zones against C6-HSL, and 3-oxo-C6-HSl with mean diameter sizes of 30 and 24, respectively. Furthermore, it maintained its activity at pH 6, 8, and 10 evidenced by the presence of inhibition zones against C6-HSL and 3-oxo-C6-HSl with mean diameter sizes of 31 and 25, respectively at pH 6 and 28, and 23, respectively at pH 8, 25, and 21, respectively at pH 10. However, the enzyme activity was lost completely when incubated at 90^∘^C for 60 min and at pH 4 evidenced by the absence of inhibition zones.

### Determination of minimum inhibitory concentration (MIC) and minimum bactericidal concentration (MBC) for ceftiofur, *N*-acyl homoserine lactonase, and ceftiofur* N*-acyl homoserine lactonase niosome using broth macro-dilution method

Our study revealed that *N*-acyl homoserine lactonase and niosome (50% v/50%v) showed antibacterial properties against tested *K. pneumoniae* strain at MIC of 0.8 and 2.4 µgml^−1^, respectively while MBC was 1.6 and 4.8, respectively. On the other hand, ceftiofur and niosome (75% v/25%v) are not effective antibacterial agents against tested *K. pneumoniae* strain KP19; their MICs were 16 and 32 µgml^−1^, respectively,

### Determination of synergy between the ceftiofur and *N*-acyl homoserine lactonase using checkerboard broth micro-dilution method

The combination of ceftiofur and *N*-acyl homoserine lactonase has a synergistic effect on the tested *K. pneumoniae* strain, as the FIC index of ceftiofur /* N*-acyl homoserine lactonase was 0.042.

### Characterization of the ceftiofur *N*-acyl homoserine lactonase niosome

**Fig. 3:**
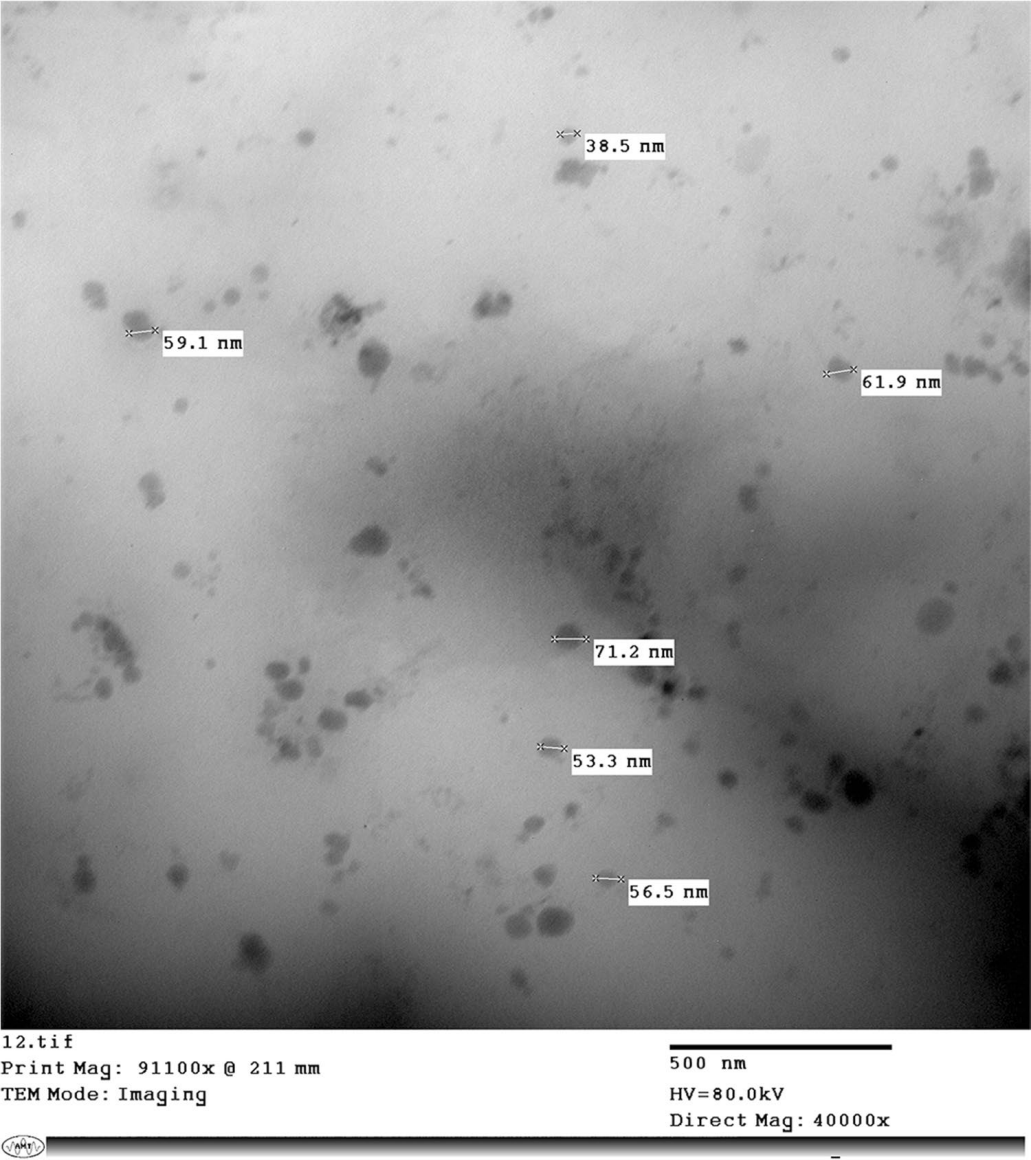
Characterization of ceftiofur *N*-acyl homoserine lactonase niosome using Transmission Electron Microscope. TEM analysis revealed that the niosome had spherical shape with an average size of 56.5 ± 4.41 nm

The niosome (50% v/50%v) was characterized for the nano size, conductivity, viscosity, polydispersity index (PDI), and zeta potential that revealed 20.54 ± 1.28 nm, 725 us/cm, 0.894, 0.27, and -17.0 mV, respectively. TEM analysis revealed that the niosome had a spherical nature with no aggregation and size homogeneity and its average size was 56.5 ± 4.41 nm (Fig. 3).

### Cytotoxicity of the ceftiofur *N*-acyl homoserine lactonase niosome

The viability results of Vero cells treated with different concentrations of niosome (0.01, 0.1, 1,10, 100 µgml^−1^) revealed 102.975 ± 2.801, 100.852 ± 1.990, 95.5193 ± 2.118, 90.6301 ± 0.815, and 1.35356 ± 0.843, respectively. The cytotoxic activity was evaluated by IC_50_ value. The calculation of IC_50_ revealed that the IC_50_ value of the niosome was > 22.62 µgml^−1^.

#### Efficacy of ceftiofur *N*-acyl homoserine lactonase niosome in the treatment of multi-resistant *K. pneumoniae* in broilers

##### Clinical signs and mortality

Groups Ӏ, Ш, and IV showed no clinical signs, while moderate to severe signs such as diarrhea, respiratory signs, ruffling feathers, and progressive weakness were recorded in groups V and П. On the other hand, group VI showed mild respiratory signs and diarrhea (Table 3).

**Table 3:** Clinical signs in the different groups of the experimental study

Group	Respiratory signs	Diarrhea	General signs	Mortality
Ӏ (Negative control group)	No signs			No mortalities
П (Positive control group)	Moderate to severe signs, including gasping and abnormal breathing on days 3 to 6 post-infection with *K. pneumoniae*	Moderate to severe diarrhea on days 2 to 6 post-infection with *K. pneumoniae*	Ruffling feathers, and progressive weakness on days 2 to 6 post-infection with *K. pneumoniae*	10% mortality on day 4 post-infection with *K. pneumoniae*
Ш (Ceftiofur-treated group)	No signs			No mortalities
IV (Niosome-treated group)	No signs			No mortalities
V (Ceftiofur-treated group challenged with *K. pneumoniae)*	Moderate to severe signs, including gasping and abnormal breathing on days 4 to 6 post-infection with *K. pneumoniae*	Moderate to severe diarrhea on days 2 to 6 post-infection with *K. pneumoniae*	Ruffling feathers, and progressive weakness on days 2 to 6 post-infection with *K. pneumoniae*	No mortalities
VI (Niosome-treated group challenged with *K. pneumoniae)*	Mild respiratory signs on days 4 to 6 post-infection with *K. pneumoniae*	Mild diarrhea on days 2 to 6 post-infection with *K. pneumoniae*	No signs	No mortalities

Group П showed 10% mortality on the fourth day post-infection compared to the other groups (Ӏ, Ш, IV, V, and VI) that revealed no evidence of mortalities (Table 3).

##### Gross lesions

No gross lesions were recorded in groups Ӏ, Ш, and IV. Groups (П and V) showed noticeable severe lesions, such as polyserositis in their internal organs, enteritis, and congestion of the lung, liver, and spleen. In contrast, mild lesions of polyserositis, enteritis, and congestion in the lung, liver, and spleen were recorded in 20–30% of chickens in group VI (Table 4).

**Table 4:** Gross lesions in the positive control group and experimental challenged groups treated with ceftiofur and ceftiofur *N*-acyl homoserine lactonase niosome

Group	Polyserositis	Lung congestion	Liver congestion	Spleen congestion	Enteritis
П (Positive control group)	27/27 (100%)	27/27 (100%)	22/27 (81.5%)	20/27 (74%)	27/27 (100%)
V (Ceftiofur-treated group challenged with *K. pneumoniae)*	30/30 (100%)	30/30 (100%)	24/30 (80%)	21/30 (70%)	30/30 (100%)
VI (Niosome-treated group challenged with *K. pneumoniae)*	6/30 (20%)	9/30 (30%)	6/30 (20%)	6/30 (20%)	9/30 (30%)

##### Enumeration of* K. pneumoniae*

There were significant differences in the *K. pneumoniae* counts in the tracheal swab samples between the positive control group (П), niosome and ceftiofur treated challenged groups (VI and V), by a Kruskal–Walli’s test (*P* = 0 000). The niosome and ceftiofur treated challenged groups (VI and V) showed a decrease in the count of *K. pneumoniae* in the tracheal swabs by 3.39 and 0.15 log_10_ CFU, respectively compared to the positive control groups (II) that showed an increase in the count by 1.92 log_10_ CFU (Table 5). There were significant differences in the *K. pneumoniae* counts between ceftiofur and niosome treated challenged groups (V and VI) by the Mann–Whitney test (*P* = 0 000).

**Table 5:** Effect of ceftiofur and ceftiofur *N*-acyl homoserine lactonase niosome treatments on the count quantification of *K. pneumoniae* in the experimental groups V and VI at the age of 16 to 21 days of the experimental study

Day	Count log_10_ (CFU/g of tracheal swabs)			
	Group П (Positive control)	Group V (Ceftiofur-treated group challenged with *K. pneumoniae*)	Group VI (Niosome- treated group challenged with *K. pneumoniae*)	
Day 16^th^	5.27 ± 0.01	5.8 ± 0.01	5.82 ± 0.02	*P*- value
Day 17^th^	6.79 ± 0.05	7.1 ± 0.03	6.14 ± 0.03	
Day 18^th^	6.89 ± 0.03	5.76 ± 0.03	5.48 ± 0.02	
Day 19^th^	7 ± 0.01	5.68 ± 0.01	4.59 ± 0.02	
Day 20^th^	7.17 ± 0.02	5.67 ± 0.01	3.59 ± 0.01	
Day 21^th^	7.19 ± 0.02	5.65 ± 0.01	2.43 ± 0.01	
Mean reduction Count log_10_	–1.92 ± 0.01^b^	0.15 ± 0.00^a^	3.39 ± 0.01^c^	0.000

Counts were expressed by mean ± standard deviation of the three replicated groups. Values of mean reduction count with different superscript letters (a, b, and c) were significantly different (*p* < 0.05)

##### Quantification of ceftiofur in serum samples

**Fig. 4:**
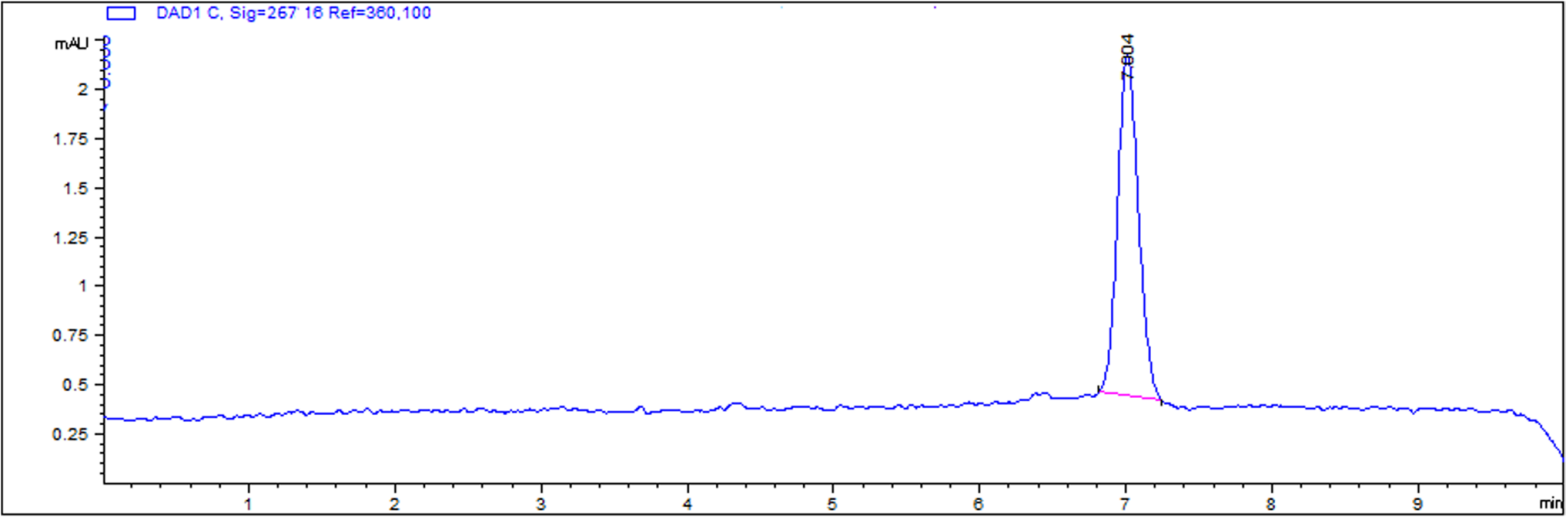
HPLC chromatogram of ceftiofur standard at a concentration of 5 µg/ml. Ceftiofur standard expressed a single sharp peak with a retention time of 7.004 min

The method was accurate with a high recovery of (93–111%) and good linearity (correlation coefficient ˃ 0.99). The detection limit and quantification limit of the method for serum were 0.01 and 0.03 μgml^−1^, respectively. Ceftiofur was detected as a single sharp peak after 7.004 min and showed no interference in the matrix (Fig. 4).

##### Pharmacokinetics results

**Fig. 5:**
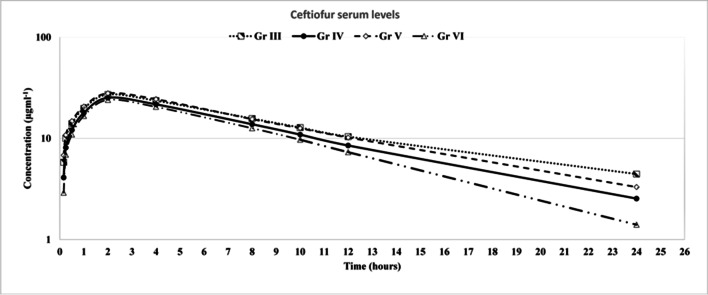
The concentration–time semilogarithmic plot for ceftiofur in serum after intramuscular administrations of ceftiofur and ceftiofur *N*-acyl homoserine lactonase niosome in groups III, IV, V, VI. The concentrations of ceftiofur and desfuroylceftiofur-related metabolites in serum following administration of ceftiofur and niosome (10 mg/kg BW I.M) were expressed as (mean ± standard deviation)

The pharmacokinetic parameters of ceftiofur administrated IN groups Ш, IV, V, and VI (10 mg/kg BW I.M) in were outlined in Table 6. There was a significant increase in the absorption half-life (t_1/2 ka_), elimination half-life (t_1/2Beta_), maximum serum concentration (C_max_), area under curve (AUC), and mean residence time (MRT) in the niosome-treated groups (IV and VI) compared with ceftiofur treated groups (Ш and V) (p < 0.05). In contrast; the total clearance of ceftiofur (Cl/F) was significantly lower in the niosome-treated groups (IV and VI) than ceftiofur-treated groups (Ш and V) (p < 0.05). Ceftiofur was quantifiable in the serum in groups Ш, IV, V, and VI at different time points after single intramuscular administration of 10 mg/kg BW (Fig. 5).

**Table 6:** Kinetics of ceftiofur after single intramuscular injection of 10mgKg^−1^ in treated non- challenged groups (Ш and IV) and treated challenged groups (V, and VI)

Kinetic Parameters	Group Ш (Non- challenged treated with ceftiofur)	Group IV (Non- challenged treated with niosome)	Group V (Ceftiofur-treated group after challenge with *K. pneumoniae*)	Group VI (Niosome-treated group after challenge with *K. pneumoniae*)
t_1/2 ka_ (h)	0.7 ± 0.9	0.98 ± 0.8*	0.59 ± 0.6	1.1 ± 0.9**
t_1/2Beta_ (h)	5.5 ± 0.15	6.8 ± 0.7*	4.9 ± 0.5	5.7 ± 0.7**
V/F (mg) (µg/ml)	0.22 ± 0.12	0.3 ± 1.2*	0.28 ± 0.7	0.22 ± 0.3
CL/F(mg)(µg/ml)/hr	0.04 ± 0.54	0.03 ± 0.9*	0.09 ± 0.8	0.04 ± 0.8**
T_max_ (h)	2.3 ± 0.7	2.4 ± 0.6	2.2 ± 0.6	2.5 ± 0.7
C_max_ (μg/ml)	24.3 ± 0.5	26.3 ± 0.8*	23.7 ± 0.8	25.4 ± 0.4**
AUC_0-24_ (μg h/ml)	247.9 ± 0.7	254.7 ± 0.9*	228.02 ± 0.7	236.8 ± 0.9**
MRT (h)	8.9 ± 0.6	10.2 ± 0.8*	8.2 ± 0.8	9.8 ± 0.7**

All values are expressed as mean ± SD, *Significant change at *p* < 0.05 with respect to Group Ш using t-test, ** Significant change at *p* < 0.05 with respect to Group V using t-test, t_1/2 ka_: absorption half-life_,_ t_1/2Beta_: elimination half-life, V/F: apparent volume of distribution, CL/F: apparent total clearance of the drug from serum, T_max_: time to reach maximum serum concentration_,_ C_max_: maximum serum drug concentration, AUC_0-24_: area under the serum concentration-time curve from time zero to time 24 hs, MRT: mean residence time

## Discussion

Antimicrobial-resistant infections pose a major threat to animal and human health as a result of antibiotic abuse or overuse, causing high mortalities (Serwecińska 2020). The emergence of extended-spectrum beta-lactamase (ESBL)-producing *K. pneumoniae* over the past few years in poultry raises critical concerns for therapies against multidrug-resistant infections (Li et al. 2022). The risk of zoonotic transmission of MDR *K. pneumoniae* from poultry to humans through direct contact or food chain is still unknown. Some investigations have reported the dissemination of ESBL- producing *Escherichia coli* from poultry to farm workers (Huijbers et al. 2014; Leverstein-van Hall et al. 2011; Tansawai et al. 2019). Antivirulence drugs have been identified as a promising option for controlling antibiotic-resistant bacteria through targeting virulence functions and behaviors rather than pathogen viability (Tang and Zhang 2014). Many macromolecular QQ agents have been discovered, but none of them have been commercialized. The greatest challenge for the commercialization of antivirulence drugs is technological and economic, since the purification of bioactive compounds is necessary for their use on a commercial scale, which is expensive to produce in enough quantities for the drug design and delivery industry (Clatworthy et al. 2007; Tang and Zhang 2014; D'Angelo et al. 2018)**.** Furthermore, current limitations on the administration of macromolecular QQ agents, such as their narrow spectrum and low bioavailability have prompted the search for new formulation options (Park et al. 2011; Tang and Zhang 2014). In this study, the QQ lactonase enzyme AhlK, produced by eight *K. pneumoniae* isolates, was formulated with ceftiofur in a niosomal form to determine its efficacy in the treatment of multi-beta lactam-resistant *K. pneumoniae* infection in broilers. Ceftiofur sodium was selected to be used as it is the most commercially available formulation of ceftiofur tested in avian species which permits once-daily treatment in mammals. Eight QQ *K. pneumoniae* isolates containing *ahlK* gene showing resistance to beta-lactam antibiotics have been recovered. According to Kusada et al. (2019), this phenotypic bi-functionality was reported in bacteria inhabiting natural ecosystems, including *P. aeruginosa* PAO1, *Acidovorax* species MR-S7, *Sphingomonas* species S1, *Acidovorax* species M2, *Acidovorax* species M6, *Diaphorobacter* species S2, *Stenotrophomonas* species S17, and *Mycobacterium* species M1. To date, there are no published reports on the isolation of AHL-degrading *K. pneumoniae* from poultry and environmental samples. The percentage of QQ isolates recovered from environmental samples in this study was 75% (6 of 8) higher than reported in previous studies of other species in soil, where percentages ranged from 2% to 4.8% (Dong et al. 2000, 2002; D’Angelo-Picard et al. 2005). Two QQ *K. pneumoniae* isolates were recovered from cloacal swabs collected from ducks; these bacteria may have been driven from the gastrointestinal tract, which could reflect the ability of bacteria to enter and colonize this ecosystem (Ghanei-Motlagh et al. 2019).

A blast search for the *ahlK* gene for six *K. pneumoniae* isolates showed high nucleotide sequence similarity (up to 99%) with other *ahlK* genes existing in the NCBI database. The selection of the six isolates was based on the high intensity of bands obtained in the PCR reaction. Similarly, a high genetic similarity, greater than 90%, was observed in AiiA lactonase recovered from the *Bacillus* genus (Sakr et al. 2013).

The combined AHLs synthesis and degradation were reported in six *K. pneumoniae* isolates in this study, which suggests that bacteria can control their own AHL production and repression depending on the growth phase (Chan et al. 2011). These results support previous studies that revealed the co-existence of AHL synthesis and degradation in *Agrobacterium tumefaciens,* marine *Shewanella,* and ginger rhizosphere *Burkholderia* GG4 strains (Zhang et al. 2002, 2004; Tait et al. 2009; Chan et al. 2011).

The QQ activity of the extracted lactonase enzyme was assessed against different synthetic AHLs using biosensor and HPLC assays. The results revealed that the selected strains can degrade C6-HSL and 3-oxo-C6-HSl, which supports earlier studies that displayed C6-HSL and 3-oxo-C6-HSL degradation by the AhlK lactonase enzyme produced by *K. pneumoniae* (Park et al. 2003; Chan 2013). The use of MOPS solution (pH 6.5) in the extraction of lactonase enzyme and during HPLC analysis was to ensure that there was no change in the pH values with time to avoid recyclization of the opening lactone ring (See-Too et al. 2017). It was observed complete degradation of C6-HSL and 3-oxo-C6-HSL in buffered aqueous media containing 0.07% NaCl compared to buffered LB medium containing 10 gl^−1^ of NaCl. This might be attributed to the higher solubility of sodium chloride in the aqueous medium than in the LB medium and the interference of yeast extract present in the LB medium with AhlK lactonase quenching activity (Dor et al. 2021). These findings suggested that AhlK lactonase might be halophilic and reflected the increased salt content of the collected litter samples, which might be related to the repeated reuse and composting of litter on the farms (Wang et al. 2016). According to Liu et al. (2019), AhlX was the first halophilic AHL lactonase isolated from the marine *Salinicola salaria* MCCC1A01339 that could tolerate 25% NaCl.

The stability of the AhlK lactonase enzyme was further assessed against different thermal and pH conditions. The lactonase enzyme showed high thermal stability; it retained 96.7% of its activity when incubated at 40^∘^C and 60^∘^C for 60 min but lost its activity when incubated at 90^∘^C for 60 min. This is consistent with previous findings of Sakr et al. (2013), who reported that the lactonase enzyme from *B. weihenstephanensis* retained > 90% of its activity when incubated at 50 °C for 60 min and lost its activity when the enzyme was incubated at 90 °C for 60 min. On the other hand, Wang et al. (2004) reported the inactivation of recombinant lactonase enzyme AiiA_240_ isolated from Bacillus species at 45^∘^C. Cao et al. (2012) reported that lactonase enzyme *Bacillus* sp. Strain AiiA _AI96_ retained about 60% of its activity after incubation for 3 min at 90^∘^C. Furthermore, the activity of the AhlK lactonase enzyme remained unchanged at pH 6, retained > 90% and 80%, of its activity at pH 8 and 10, respectively, and completely lost its activity at pH 4. Similarly, previous studies have displayed high stability of AHL lactonase enzymes over a pH range of 6–9 and completely lost their activity at acidic pH (Wang et al. 2004; Cao et al. 2012; Sakr et al. 2013).

Two nano-liposomal formulations were evaluated to select the most suitable one, enabling efficient quenching activity and delivery of ceftiofur *N*-acyl homoserine lactonase. The results revealed that niosome (50% v/50%v) had antibacterial properties against the tested *K. pneumoniae KP*19 isolate with a MIC value 13-fold lower than obtained using ceftiofur alone. Furthermore, there were synergistic interactions between ceftiofur and the AhlK lactonase enzyme, as indicated by the FIC index. It was reported that beta-lactam antibiotics were one of the most antimicrobial groups associated with a positive interaction potential (Haroun and Al-Kayali 2016). Bortolotti et al. (2019) reported that the combination of LasR quorum sensing inhibitors with ciprofloxacin could reduce the formation of biofilm and the antibiotic tolerance of *P. aeruginosa*. According to (Vinoj et al. 2015), the AiiA lactonase loaded on gold nanoparticles has a promising antibiofilm activity against multi-drug resistant *Proteus* species. Gupta and Chhibber (2019) reported that the AiiA lactonase loaded on silver nanoparticles has a significant effect on the reduction in exopolysaccharide production, cell surface hydrophobicity, and metabolic activity, and has promising anti-biofilm activity against multidrug-resistant *K. pneumoniae*. Tween 20 was selected to be used as a non-ionic surfactant in the niosome formulation due to its higher stability, compatibility, solubility, and lower toxicity compared to anionic and cationic counterparts (Kumar and Rajeshwarrao 2011). Sonication was used during niosome preparation to obtain vesicles with homogenous size distribution and no aggregation (Hallaj-Nezhadi and Hassan 2015); this allowed the mean diameters of niosome to be reduced from 528 nm to 20.54 ± 1.28 nm.

The niosome (50% v/50%v) was further characterized using TEM and zetasizer. According to Ag Seleci et al. (2016), niosomes were divided into three types based on their sizes and bilayer composition: small unilamellar vesicles, which range in size from 10 to 100 nm, large unilamellar vesicles which range in size from (100–3000 nm), and multilamellar vesicles, which include several bilayers. In this study, TEM analysis revealed that the prepared niosome was a spherical small unilamellar vesicle with no aggregates. Previous studies displayed that AiiA Lactonases loaded on gold and silver nanoparticles were spherical and their sizes ranged from 10–30 nm (Vinoj et al. 2015; Gupta and Chhibber 2019). Zeta potential is an important parameter that can influence the stability, pharmacokinetics, bio-distribution, and toxicity of niosomes (Ge et al. 2019). In this study, the niosome had a high negative charge, which leads to an increase in its stability for longer periods and makes it less likely to aggregate (Ge et al. 2019).

The cytotoxicity of niosome (50% v/50%v) was investigated in Vero cell lines. The results revealed the absence of cytotoxic effects at concentrations of 0.01, 0.1, 1, and 10 μgmL^−1^ but the cell viability was affected at 100 μgmL^−1^. This is consistent with a previous study by Vinoj et al. (2015) who reported that the treatment of a mouse macrophage cell line with AHL lactonase coated on gold nanoparticles isolated from Bacillus licheniformis (AiiA AuNPs) at concentrations of 2 to 8 M did not have a cytotoxic effect but still had an impact on cell viability at concentrations higher than 8 M. According to Gupta and Chhibber (2019), AHL Lactonase coated on silver nanoparticles (AiiA AgNPs) isolated from Bacillus sp. ZA12 did not have a cytotoxic effect on mouse macrophage cells at concentrations of 1.5, 3, and 6 µg/mL but still had a great effect on cell viability at concentrations of 50 gmL^−1^, 75 gmL^−1^, and 100 gmL^−1^.

The potential use of ceftiofur *N*- acyl homoserine lactonase niosome in treating multi-resistant *K. pneumoniae* in broilers was evaluated. The selection of the intramuscular route for the delivery of the formulated niosome was based on the results of previous studies, which reported that ceftiofur is poorly absorbed after oral administration and the delivery of proteins after oral administration is hindered by proteolytic enzymes, pH gradients, and the epithelial barrier (Deghmane et al. 2016; Haddadzadegan et al. 2022). Cao et al. (2012) reported that *N*-acyl homoserine lactonase AiiAB546 was unable to protect zebrafish against *Aeromonas hydrophila* infection by oral administration. The present results reported the effectiveness of ceftiofur *N*-acyl homoserine lactonase niosome in controlling mortalities in the niosome-treated birds in groups (IV and VI). This result is consistent with previous studies that revealed the ability of recombinant *N*-acyl homoserine lactonase Ahl-1and the combination of lactonase with ciprofloxacin to reduce mortality in murine models (Gupta et al. 2015; Sakr et al. 2021).

Furthermore, it was found that the largest reductions in the *K. pneumoniae* count in tracheal swab samples were in group VI on day 21 of the study period following the administration of ceftiofur *N*-acyl homoserine lactonase niosome for five consecutive days. According to Sakr et al. (2021) employing a mouse model, topically applied recombinant *N*-acyl homoserine lactonase Ahl-1 reduced *P. aeruginosa* count in blood, lung, and liver by 4 logs after three days of treatment. Gupta et al. (2015) reported that topical application of the combination of lactonase and ciprofloxacin in a murine model revealed the reduction of the *P. aeruginosa* load in blood, lung, and liver by a range of 1.5 to 2.4 logs after three days of treatment.

Certain pharmacokinetic parameters were studied for establishing a rational dosage schedule that achieves the desired therapeutic outcome. The serum concentration of ceftiofur can be quantified by measuring desfuroylceftiofur, the active metabolite of ceftiofur, which is produced by cleaving the thioester bond (CVMP 1999). Ceftiofur's antimicrobial properties are time-dependent, meaning the bactericidal activity is determined by the duration of exposure to the drug over MIC (Hooper et al. 2016). Depending on this fact, the detected serum ceftiofur concentration in the non-challenged group Ш was less than MIC (16 µg/ml) at 8 h following administration of ceftiofur at a dose of 10 mg/kg BW I.M in broilers. These findings suggest the mandatory repetition of ceftiofur every 8 h which would be inconvenient and impractical in the treatment of *K. pneumoniae*. On the contrary, the ceftiofur serum concentration fell below the detected MIC (2.4 µgml^−1^) in the non-challenged group IV at 24 h following administration of niosome at a dose of 10 mg/kg BW in broilers. As a result, the niosome is the most convenient treatment regimen for *K. pneumoniae*. The obtained serum levels of ceftiofur in the niosome-treated groups (IV and VI) were lower than those obtained in the ceftiofur-treated groups (Ш and V). This might be attributed to the higher penetrating power of niosome in the tissues. These results support a previous study that showed the great efficacy of nano-antimicrobials against infectious bacteria (Agnihotri and Dhiman 2017).

The t_1/2 ka_, t_1/2Beta_, C_max_, AUC, and MRT findings of the present study were higher in the niosome treated groups (IV and VI) compared to ceftiofur treated groups (Ш and V). This might be attributed to the slow release of drugs from liposome molecules (Akbarzadeh et al. 2013)**.** The long elimination half-life (t_1/2 ßeta)_ reported in the niosome-treated groups (IV and VI) (6.8 ± 0.7 and 5.7 ± 0.7 h, respectively) reflected its high absorption and low clearance time (0.03 ± 0.9 and 0.04 ± 0.8), respectively (Jackson et al. 2012). The maximum serum concentration (Cmax) of ceftiofur in tested groups (Ш, IV, V, and VI) was observed after 2 h following administration of ceftiofur and niosome. This is in line with the fact that maximum blood concentrations of ceftiofur and metabolites were achieved within 0.5 and 2 h of dosing (CVMP 1999). In addition, the Cmax, Tmax, and t_1/2ß_ values of ceftiofur in the non-challenged ceftiofur treated group were 24.3 ± 0.15, 2.3 ± 0.7, and 5.5 ± 0.15, respectively. This is in line with the findings of (El-Sayed et al. 2015)**,** who reported that a C_max_ of 25.94 μgml^−1^ achieved at a maximum time of t_max_ = 2.51 h with 5.47 h as t1/2 ß value.

## Conclusions

Finally, we conclude that the encapsulation of ceftiofur *N*-acyl homoserine lactonase in a niosomal formulation provided higher efficacy in the treatment of multi-resistant *K. pneumoniae* infection in broilers. Furthermore, this study provided new insight into the diversity of organisms that display both multiple beta-lactam antibiotic resistance and QQ activities, as well as quorum sensing and quorum quenching activity. Further studies on the correlation between beta-lactam antibiotic resistance and QQ activities and the salt tolerance of QQ molecules are needed.

### Supplementary Information

Below is the link to the electronic supplementary material.

Fig. S1 Pairwise identity matrix of nucleotide and amino acid sequences of six QQ *K. pneumoniae* isolates recovered from duck. The pairwise analysis of the six QQ *K. pneumoniae* isolates recovered from duck and other related isolates was based on sequencing of the *ahlK* gene. (JPG 372 KB)

Supplementary file2 (DOCX 13 KB)

## Data Availability

The authors confirm that the data supporting the findings of this study are available within the article and its supplementary information.

## Code availability

Non-applicable.
