# Supplementary figures and images for: Efficacy of ceftiofur N-acyl homoserine lactonase niosome in the treatment of multi-resistant Klebsiella pneumoniae in broilers
Source: Vet Res Commun. 2023 Jul 10;47(4):2083–100. doi: 10.1007/s11259-023-10161-7 (PMC10697884; doi:10.1007/s11259-023-10161-7)

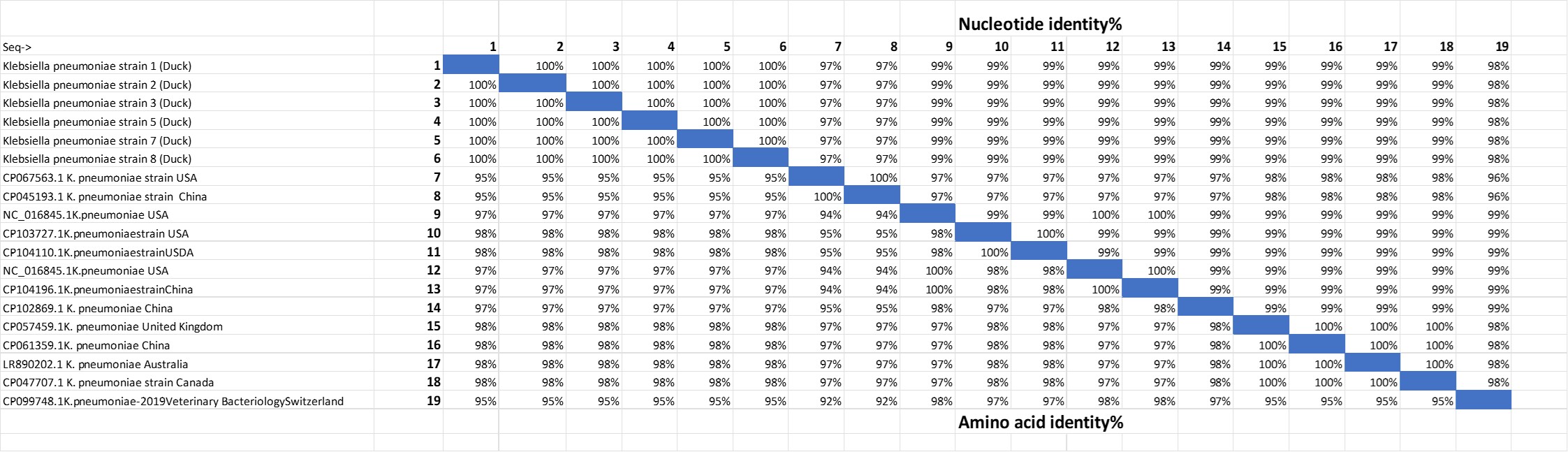

Supplement: Supplementary file 1 — Fig. S1 Pairwise identity matrix of nucleotide and amino acid sequences of six QQ K. pneumoniae isolates recovered from duck. The pairwise analysis of the six QQ K. pneumoniae isolates recovered from duck and other related isolates was based on sequencing of the ahlK gene. (JPG 372 KB) [file 11259_2023_10161_MOESM1_ESM.jpg]
