# Supplementary material for: Efficacy of ceftiofur N-acyl homoserine lactonase niosome in the treatment of multi-resistant Klebsiella pneumoniae in broilers
Source: Vet Res Commun. 2023 Jul 10;47(4):2083–100. doi: 10.1007/s11259-023-10161-7 (PMC10697884; doi:10.1007/s11259-023-10161-7)
Supplement: Supplementary file 2 — Supplementary file2 (DOCX 13 KB) [file 11259_2023_10161_MOESM2_ESM.docx]

**Table S1. Type of samples collected from different groups of the experimental study**

| **Group** | **Tracheal swabs** | **Blood samples** | **Organs** |
| --- | --- | --- | --- |
| Ӏ  (Negative control group) |  |  | Lung, liver, spleen, and intestine samples were taken on day 21 from birds of all groups after euthanizing to examine gross lesions |
| П  (Positive control group) | Tracheal swabs were collected on days 16–21 for the counting of K. pneumoniae |  |  |
| Ш  (Ceftiofur-treated group) |  | Blood samples were collected from the wing veins of five birds at nine-time points (0.16, 0.5, 1, 2, 4, 8, 10, 12 and 24 hours) following administration of ceftiofur and niosome on day 16 for studying of pharmacokinetic (PK) parameters. |  |
| IV  (Niosome-treated group) |  |  |  |
| V  (Ceftiofur-treated group challenged with  *K. pneumoniae)* | Tracheal swabs were collected on days 16–21 for the counting of K. pneumoniae |  |  |
| VI  (Niosome-treated group challenged with  *K. pneumoniae)* |  |  |  |
